# Residence time in drug discovery: current insights and future perspectives

**DOI:** 10.1007/s43440-025-00748-z

**Published:** 2025-06-09

**Authors:** Szymon K. Kordylewski, Ryszard Bugno, Sabina Podlewska

**Affiliations:** Maj Institute of Pharmacology Polish Academy of Sciences, Smętna 12, Kraków, 31-343 Poland

**Keywords:** Residence time, Molecular dynamics, Ligand kinetics, G protein-coupled receptors

## Abstract

The temporal stability of ligand-receptor complexes is increasingly acknowledged as a critical factor in drug discovery, influencing both efficacy and pharmacodynamics. Although the relationship between the duration of compound action and complex stability can be traced back to Paul Ehrlich’s 19th-century doctrine *Corpora non agunt nisi fixata*, its significance has gained renewed attention in recent years. This review comprehensively examines the concept of residence time (RT). We first summarize key ligand binding models (lock-and-key, induced-fit, and conformational selection) and delve into various perspectives on how RT impacts functional outcomes. Furthermore, we discuss experimental methods for measuring RT, highlighting both radioligand and non-radioligand approaches. The growing interest in RT has spurred advancements in computational techniques, particularly molecular dynamics simulations, which utilize diverse strategies to observe dissociation events. We outline these molecular dynamics-based methods, their theoretical foundations, and provide examples of their application in assessing RT. Finally, we highlight molecular determinants of prolonged RT, focusing primarily on G protein-coupled receptors (GPCRs) while also incorporating relevant data from other receptor classes.

**Graphical Abstract:**

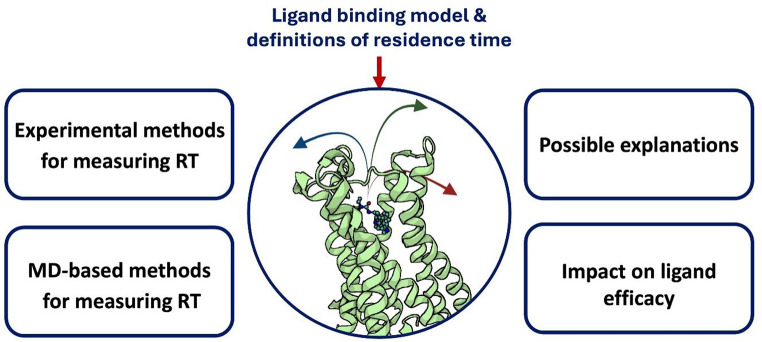

## Introduction

The duration of ligand-target complex existence has been largely neglected in past studies on ligand-receptor interactions. Historically, research focused on bioactive compounds primarily revolved around determining thermodynamic constants that characterize ligand affinity and functional activity at receptors. While early efforts to investigate binding kinetics trace back to the early 20th century^1^ it is only in recent years that kinetic considerations have gained significant attention.

Commonly used thermodynamic parameters include the dissociation constant (*K*_*D*_), inhibition constant (*K*_*i*_), half-maxi mal inhibitory concentration (*IC*_*5*__0_), or half-maximal effective concentration (*EC*_*50*_). Although these parameters provide valuable insights into ligand affinity, their predictive power concerning drug efficacy is limited. In vivo, the occurrence of ligand-receptor interactions also hinges on the amount of ligand that reaches the receptor proximity, which is influenced by a multitude of dynamic factors, including absorption, distribution, metabolism, and excretion (ADME) [2, 3]. These processes continuously modulate ligand concentrations at the target site, presenting a challenge to ensure adequate ligand availability. It is noteworthy that insufficient efficacy is estimated to account for up to 66% of drug failures in Phase II and Phase III clinical trials [4–7].

To enhance predictions of drug efficacy, researchers are increasingly incorporating parameters beyond traditional ADME properties. While conventional affinity-based approaches provide valuable insights into the potential ligand-receptor complexes formation, they rely primarily on equilibrium-state measurements conducted under controlled laboratory conditions. However, these may not adequately reflect the transient and dynamic nature of receptor-ligand interactions in vivo. Consequently, contemporary research is shifting toward identifying novel predictors that extend beyond affinity, integrating kinetic and mechanistic insights related to ligand binding and unbinding dynamics to improve translational success.

This review systematically explores the concept of the ligand-receptor complex duration and its increasing relevance in drug discovery. It discusses experimental and computational methodologies for assessing this parameter and outlines the molecular features associated with prolonged binding, with particular emphasis placed on GPCRs.

## Definitions

**Fig. 1:**
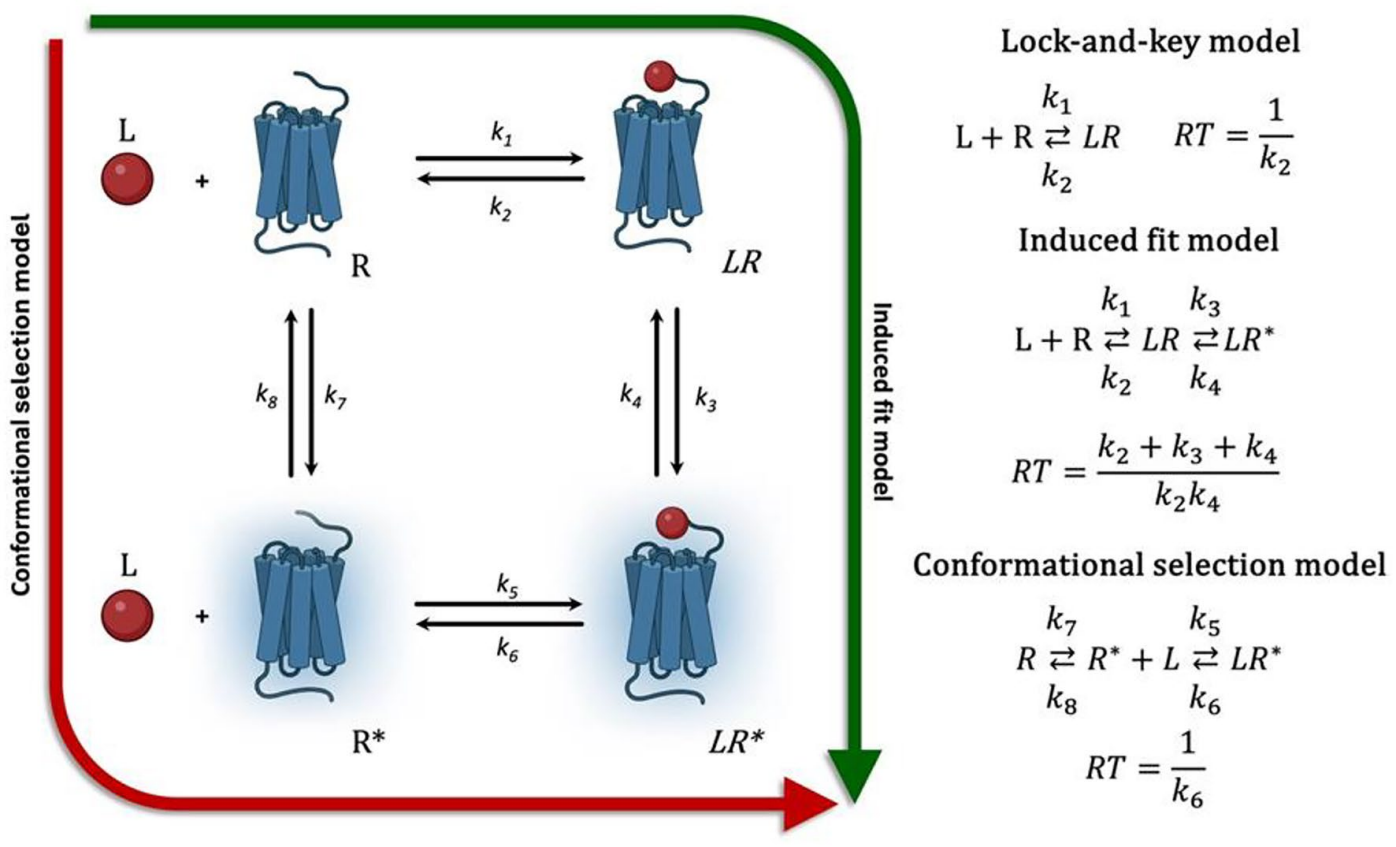
Schematic representation of an agonist binding to a GPCR in different models [14]. The induced fit model posits that the ligand (L) initially binds to the inactive receptor conformation (R), forming an inactive LR complex, which subsequently undergoes a conformational transition into the active LR* complex, capable of G protein activation. In contrast, the conformational selection model proposes that the receptor exists in an equilibrium between an inactive (R) and an active (R*) conformation, with the ratio of these states in the native system determining the receptor’s basal activity. An agonist ligand preferentially stabilizes the active conformation (R*), an inverse agonist inactive conformation (R), and a neutral antagonist exhibits equal affinity for both the inactive (R) and active (R*) receptor conformations, thereby maintaining the equilibrium between the two states. The earlier lock-and-key model does not account for conformational changes and can be described as the direct association of L and R to form the LR complex; *k*_*1…8*_ denote reaction rate constants

The binding of a ligand to a receptor is commonly conceptualized through three primary models, each grounded in distinct mechanistic assumptions (Fig. 1). The earliest and most straightforward is Fischer’s lock-and-key model [8], which conceptualizes the formation of the active ligand-protein complex as a simple first-order process. In this model, a small-molecule ligand (L) binds to the protein’s binding pocket (R) through mutual complementarity, such as steric fit and electronic effects, resulting in the formation of a stable ligand-protein complex (LR). This interaction leads to establishment of an equilibrium state, where the ratio of unbound ligand and protein to the complex remains constant and is defined as *K*_D_. From a kinetic perspective, the association rate constant, *k*_*on*_ (denoted as k_1_ in Fig. 1), governs the speed at which the LR complex forms, while the dissociation rate constant, *k*_*off*_ (k_2_ in Fig. 1), dictates the breakdown of the LR complex. At equilibrium, the relationship between *k*_on_ and *k*_off_ defines the previously stated K_D_, which is expressed as the ratio: *K*_*D*_=*k*_*off*_/*k*_*on*_. RT is defined as the inverse of *k*_*off*_, reflecting the duration for which the ligand-protein complex remains intact from initial formation to dissociation.

The more nuanced induced-fit model, first introduced by Koshland (1958) in the context of enzyme catalysis [9], portrays ligand binding as a process in which an initially inactive conformation of the macromolecule undergoes a structural rearrangement upon ligand association. This concept was subsequently extended to receptors, where ligand binding induces a conformational shift from an inactive receptor state (R) through an intermediate ligand-receptor complex (LR) to an active state (LR*). This sequential mechanism not only captures the dynamic nature of receptor behavior but also introduces additional kinetic steps into this process. Within this framework, RT is mathematically represented by the equation (which is reciprocal of *k*_*off*_) *RT = (k*_*2*_ *+ k*_*3*_ *+ k*_*4*_*) / (k*_2_* k_*4*_*)*, where*k*_*2*_ denotes the dissociation of the inactive complex (LR), k_3_ indicates the transition rate to the active conformation (LR*), and *k*_*4*_ represents the dissociation rate of the active complex [10]. The induced-fit model not only emphasizes the principle of mutual complementarity between a ligand and its protein target—supported by biological evidence—but also conceptually separates two critical parameters: affinity, which pertains to the formation of the LR complex, and efficacy, which refers to the LR complex’s capacity to convert into the active LR* complex that elicits a biological response. However, it is important to note that these parameters, affinity and efficacy, alone do not fully account for the observed responses in the system. The overall system behavior is also shaped by several additional factors, such as the intrinsic activity of the target, the expression level and availability of receptors for ligand binding, and the existence of spare receptors [11].

The third mechanism, widely referred to as the conformational selection model [12], proposed that the ligand selectively binds to one of the receptor’s pre-existing conformational state, which may be either active (R*) or inactive (R), depending on the nature of the ligand. Unlike the induced-fit model, this perspective posits that the receptor exists in a dynamic equilibrium between the active (R*) and inactive (R) states even before ligand binding occurs. The concept of constitutive receptor activity, introduced by Costa and Herz in 1989 [13], further extends this ideaby describing receptors that can adopt its active conformation (R*) and initiate signaling even in the absence of a ligand. Agonists preferentially bind to and stabilize the receptor’s active state (R*), effectively shifting the equilibrium toward activation in accordance with the Le Chatelier–Braun principle. In contrast, inverse agonists preferentially bind to and stabilize the inactive conformation (R), leading to a decrease in basal receptor activity. Antagonists, on the other hand, maintain the receptor’s constitutive equilibrium, effectively preserving the natural equilibrium between active and inactive receptor states. Within this framework, the RT is defined as the inverse of the dissociation rate constant *k*_*6*_, which governs the disassembly of the active receptor-ligand complex (LR*) [10].

The induced fit and conformational selection models are now widely regarded as interconnected concepts. It is now proposed that a ligand shows preference for either the inactive (R) or active (R*) receptor conformation, inducing conformational changes upon forming an active complex. A particularly illustrative case of this mechanistic interplay is the phenomenon of biased agonism, where a ligand selectively stabilized receptor conformations that favor specific intracellular signaling pathway over others [15, 16]. Structural studies employing X-ray crystallography and, more recently, cryo-electron microscopy have demonstrated that these biased effects arise from the stabilization of specific receptor conformations, which in turn facilitate the selective recruitment of specific signaling effectors, such as G proteins or β-arrestins. This observation supports the notion that receptors exist in an ensemble of conformations, of which only the most thermodynamically favorable – those residing at local minima of Gibbs free energy – are typically captures in structural snapshots.

These states form what can be described as an “energy cage,” bounded by activation energy barriers that control the transitions between stable conformations of the receptor-ligand complex. Interestingly, it has been demonstrated that a physical cage trapping the ligand within the target’s binding pocket can occur due to the so-called flap closing mechanism [17–21]. Following initial binding, the protein may undergo conformational rearrangements that create steric hindrance, effectively obstructing the ligand’s exit. A well-characterized example is the presence of an active site “lid” that acts as a dynamic gate regulating ligand dissociation. Escaping from such a trap requires overcoming energy barriers, necessitating release from the proposed “energy cage.” The potential impact of such cages on RT will be discussed in detail later in this article.

In a physiological context, a drug exerts its pharmacological effect only while being bound to its receptor. While thermodynamic constants provide insight into whether a ligand will bind to the receptor, they do not capture the quality or duration of that binding. Once introduced into the body, the ligand undergoes a sequence of pharmacokinetic processes, including absorption into the plasma, transport to the vicinity of the target receptor (often conceptualized as a single-compartment model), and eventual elimination. These processes are typically characterized by parameters that define the ligand’s bioavailability, primarily the kinetic constants of influx *k*_*in*_ and efflux *k*_*out*_, which themselves encompass multiple stages [22]. Typically, the timeframes for these pharmacokinetic processes are measured in hours, during which the ligand’s binding to the target (as determined by the *k*_*on*_) is considered nearly instantaneous. It was not until the pivotal work of Copeland et al. in 2006 [2] that the focus shifted to the duration of the active ligand-receptor complex. The authors posited that the most crucial parameter for characterizing this duration is the *k*_*off*_, the inverse of which defines the RT of the LR* complex. Within this framework, the association time is so brief compared to the dissociation process that it can be effectively disregarded [2].

Copeland et al. further outlined three key points that justify the disregard for *k*_*on*_ with reference to RT [2]. First, the upper limit of *k*_*on*_ is constrained by the diffusion rates of both the ligand and receptor under physiological conditions, setting a theoretical ceiling on its magnitude (approx. 10^9^ M^− 1^s^− 1^) [23, 24]. Secondly, *k*_*on*_ is influenced by ligand concentration; thus elevated concentrations can compensate for slower association kinetics. Finally, the dynamic behavior of ligands in vivo, characterized by processes such as redistribution and diffusion, causes variations in their local concentrations, complicating the interpretation of *k*_*on*_. While modeling receptor exposure to varying ligand concentrations presents significant challenges due to the inherent complexities of the in vivo environment, *k*_*off*_ provides a simpler and more direct parameter to study. This makes *k*_*off*_ an essential metric for evaluating drug-receptor interactions and optimizing pharmacodynamic outcomes.

With the growing recognition of compound RT as a critical parameter in drug design, several review articles have emerged summarizing the current state of the art. Some of these focus on translating RT into in vivo pharmacological effects [25–27]. Others concentrate on methodological aspects, discussing both experimental and computational approaches to measuring and estimating RT [28]. Additionally, updated theoretical frameworks and mechanistic insights into the RT concept have been addressed [29], alongside reviews highlighting in silico tools for predicting kinetic profiles of drug candidates [30].

In this review, we approach the topic of RT from first principles, aiming to provide a clear and accessible explanation of both the theoretical basis of ligand–receptor binding and the range of methods used to study this phenomenon. We offer a comprehensive overview of experimental and computational strategies, incorporating the most recent methodologies and findings. Particular emphasis is placed on the structural and physicochemical factors that govern compound RT.

### Impact of RT on ligand efficacy – selected examples

The relationship between RT and ligand efficacy remains incompletely understood, though various studies highlight the potential relevance of compound RT in pharmacological activity. For example, RT has been identified as a more reliable predictor of in vivo antibacterial activity compared to other parameters [31, 32]. Additionally, a similar positive correlation between RT and functional efficacy (but not affinity) was noted among a series of adenosine A_2A_ receptor agonists, as demonstrated using the xCEL-Ligence and cAMP assays [33]. Among potent prostaglandin DP2 receptor (CRTh2) antagonists, LAS191859 stands out due to its slow dissociation kinetics with an RT of approximately 22 h and prolonged activity in both in vitro and in vivo models [34]. The correlation between steroidogenic efficacy for Translocator Protein 18 kDa (TSPO) ligands and RT was significantly better than the correlation of efficacy with binding affinity [35–37]. Further evidence supporting the pharmacological relevance of compound RT comes from studies on TSPO ligands, where compounds with varying RTs (17–141 min) showed a clear relation between RT and the intensity of anxiolytic effects [38]. Similarly, for soluble epoxide hydrolase (sEH) inhibitors, compounds with prolonged RT exhibited improved pharmacokinetic profiles and ehnanced efficacy in a rat model of diabetic neuropathic pain [39]. Subsequent studies on sEH inhibitors with similar *K*_*i*_ values but varying RTs demonstrated that drug-target RT affects the duration of in vivo drug-target binding [40]. In the case of inflammatory protein complement C5a receptor antagonists, a longer RT has also been shown to positively influence in vivo efficacy [41]. Comparable trend was observed for a series of sulfonamide-based Na_V_1.7 antagonists, where compounds with a long RT produced more pronounced analgesic effects in vivo [42]. Moreover, RT has also been shown to be controllable with high precision using light, as demonstrated by the use of photoswitchable azo-ligands targeting vasopressin V_2_ receptors [43].

Nonetheless, the positive correlation of prolonged RT and improved efficacy is not universally consistent. For instance, when investigating muscarinic M_3_ receptor agonists, it was suggested that RT alone may not be a definite predictor of compound efficacy While some relationship between RT and pharmacological effect appears to exist, establishing a clear correlation requires testing larger compound libraries [44]. Interestingly, previous studies on tiotropium suggest that a longer RT may enhance selectivity for muscarinic M_1_ (RT = 14.6 h) and M_3_ (RT = 34.7 h) receptors over M_2_R (RT = 3.6 h) [45] and may improve in vivo efficacy [46]. In a series of adenosine A_1_ receptor agonists with nanomolar affinity (1.9–75 nM), no correlation between affinity and RT (which varied from 1.2 to 63.8 min) was observed. Interestingly, it was demonstrated that structural modifications can independently alter RT and affinity [47]. A similar lack of correlation was reported for cannabinoid CB_2_ receptor agonists: compounds with longer RTs (32–72 min) did not display better efficacy in functional in vitro assays compared to agonists with shorter RTs [48].

**Fig. 2:**
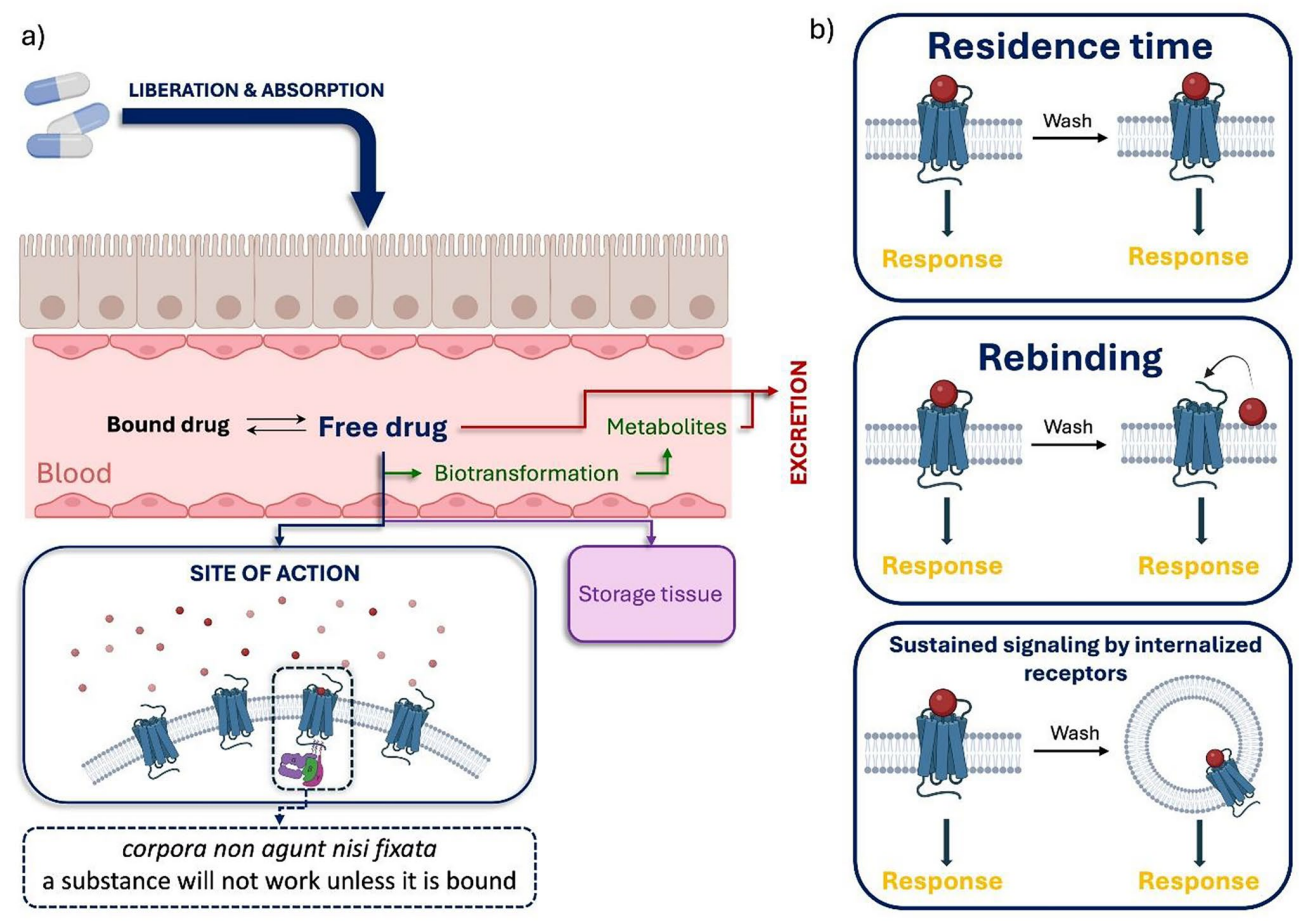
**a**) Role of RT within the LADME Profile [58]. After drug administration, the active compound can undergo a series of processes, including release from its pharmaceutical formulation, absorption into the bloodstream, and conversion into its pharmacologically active form. Once in circulation, the free drug fraction can be subject to plasma protein binding, tissue distribution, metabolism, and excretion. A portion of the active drug reaches its target site, where it binds—in this case, a G protein-coupled receptor—initiating the pharmacological effect **b**) Key mechanisms underlying sustained signaling and prolonged pharmacological response, as evidenced by in vivo studies in which rapid ligand washout following receptor saturation revealed persistent target engagement and downstream signaling. These include RT, where extended target engagement leads to prolonged pathway activation; rebinding, observed in ligands with rapid binding kinetics; and signal propagation through internalized receptors, allowing intracellular signaling to persist beyond initial receptor-ligand interaction; the figure was prepared based on [49]

In the context of GPCRs, it is important to consider additional mechanisms beyond RT that may sustain prolonged receptor functional responses. These include, for instance, rebinding, where a ligand, after dissociating from the receptor, rapidly reattaches, thereby effectively extending the functional response [49]. In such scenario, *k*_*on*_ value facilitates rapid rebinding [50] (Fig. 2). As discussed by Vauquelin et al. [51], a rapid k_on_ may correlate with a faster clinical onset of action, although this relationship is context-dependent and may not hold true across all drug classes. Conversely, Folmer cautions against overemphasizing RT as a sole determinant of efficacy, arguing that k_on_ and the dynamic interplay between association and dissociation kinetics need to be considered to fully understand drug–target interactions [52]. Another proposed mechanism is the exosite model, where one part of the ligand binds stably to the receptor while another region, responsible for activation, intermittently interacts with the binding pocket. This scenario can be likened to fishing: the angler (the portion stably bound to the receptor) casts a line (the activating part) into the lake (the receptor) at regular intervals. Prolonged signalling can also arise from internalized receptors, where the functional response persists despite the receptor being located in an endosome [49, 53, 54]. Interestingly, this process has sometimes been linked to the phenomenon of RT. For ligands with slow dissociation rates, achieving sustained signaling from internalized receptors may be more feasible. Interestingly, in some cases, this sustained intracellular activity has been associated with the phenomenon of prolonged RT [43, 55–57] (Fig. 2).

### Experimental methods for measuring ligand RT

Experimental in vitro approaches for assessing RT can be classified based on various criteria, one of which differentiates between radioligand-based and non-radioligand-based methods. Selected techniques from each group of methods are summarized below.

#### Radioligand-based strategies

One of the simplest methods for studying ligand kinetics is radiolabeling, allowing for the direct determination of association and dissociation kinetics. However, the broader application of this technique is limited by the complexity and high cost associated with the synthesis of radiolabeled ligands. Despite these limitations, radiolabeling has successfully been applied in kinetics studies involving ligands such as as, [³H]ipratropium, and [³H]tiotropium for M_2_ and M_3_ receptors [59], [^3^H]CV-11,974 (candesartan) for AT_1_ receptors [60], [^3^H]olodaterol for β_2_-adrenergic receptors (β_2_AR) [61] and a morpholine acetal derivative for NK_1_ receptors [62]. Nevertheless, the technical and economic challenges associated with synthesizing custom radiolabeled compounds have driven the development of alternative approaches that utilize readily available radioligands, eliminating the need to radiolabel the ligand of interest.

One effective way to assess RT involves competitive binding studies with a radioligand, utilizing the Motulsky and Mahan method. In such assays, receptors are simultaneously incubated with a defined concentration of the ligand of interest and the radioligand. As the LR complex forms, the emitted radioactivity increases until equilibrium is reached, where $\:{k}_{on}\left[R\right]\left[L\right]={k}_{off}\left[RL\right]$. By analyzing binding curves for different time points, it is possible to calculate *k*_*on*_, *k*_*off*_ and *K*_*D*_. Dissociation kinetics are typically assessed by rapidly disrupting the ligand-receptor equilibrium. Standard techniques include addition of a large excess of an unlabeled competitive antagonist, centrifugation followed by resuspension ina buffer, or rapid dilution with a large buffer volume.

#### Non-radioligand approaches

Radioligand-based methods have long been a fundamental tool in studying ligand-receptor interactions; however, the use of radioactive isotopes poses challenges related to safety, high costs, and the limited shelf life of labeled compounds. To address these challenges, non-radioligand methods for studying ligands —many of which are capable of providing kinetic information—have been developed, offering novel possibilities for exploring molecular interactions. Some of their representatives are described below.

The time-resolved fluorescence resonance energy transfer (TR-FRET) technique leverages non-radiative energy transfer between two fluorophores, molecules capable of absorbing electromagnetic radiation and emitting light at a different wavelength. In FRET, the donor fluorophore (protein), upon excitation occurring upon ligand binding, transfers energy to the acceptor (ligand), which results in a measurable fluorescence signal. To determine *k*_*on*_, a constant concentration of fluorescently labeled protein is used while varying the ligand concentration and monitoring FRET efficiency over time, with the slope of the observed rate providing the *k*_*on*_ value. Conversely, *k*_*off*_ is assessed by observing the decline in FRET signal after ligand removal and fitting the data to a first-order exponential decay equation to analyze dissociation. Lanthanide chelates or cryptates, such as europium or terbium, are commonly used as donor labels due to their long fluorescence lifetimes and sharp emission peaks. While ligand labeling via FRET is generally less expensive than radiolabeling, it may alter the physicochemical properties of the compound, potentially affecting binding characteristics. A modification of FRET, incorporating time-resolved fluorescence (TRF) and delayed detection (after excitation), minimizes short-lived background interference from proteins and other sources. This approach has been applied to kinetics studies of such receptors as β_2_-AR [63], A_2A_ adenosine receptor [64], histamine receptor H_1_ [65], cyclin-dependent kinase 2, bromodomain-containing protein 4 [66], and histone methyltransferase EZH2 [65].

A related method, BRET (bioluminescence resonance energy transfer), relies on bioluminescence, where the donor is a luminescent molecule, such as luciferase (e.g., NanoLuc^®^), and the acceptor is a fluorophore (e.g., AlexaFluor, Venus) [67, 68]. Advances in this method, particularly with the introduction of NanoLuc luciferase derived from the deep-sea shrimp *Oplophorus gracilirostris*, have enabled the development of NanoBRET. This technique facilitates binding and kinetic studies, which were previously infeasible due to limitations of older luciferases, such as Renilla luciferase derived from the sea pansy *Renilla reniformis* [69]. Notably, NanoBRET enables real-time kinetic binding studies not only in isolated tissues or lysates but also in live cells. This approach has been applied to receptors such as histaminergic (H_1_, H_2_, H_3_, H_4_) [70–72], muscarinic (M_2_) [73], β_2_-AR, adenosine A_1_ and A_3_ [68, 74], relaxin/insulin-like family peptide receptor 1 [75], smoothened protein [76], and ADGRG6 adhesion G protein-coupled receptor G6 [77]. An innovative application of BRET involves tagging GPCRs with NanoLuc not at the N-terminus but at the extracellular loop 2 (ECL2). This strategy has been implemented for angiotensin II receptor type 1 (AT_1_R) and the M_1_ muscarinic acetylcholine receptor [78].

**Fig. 3:**
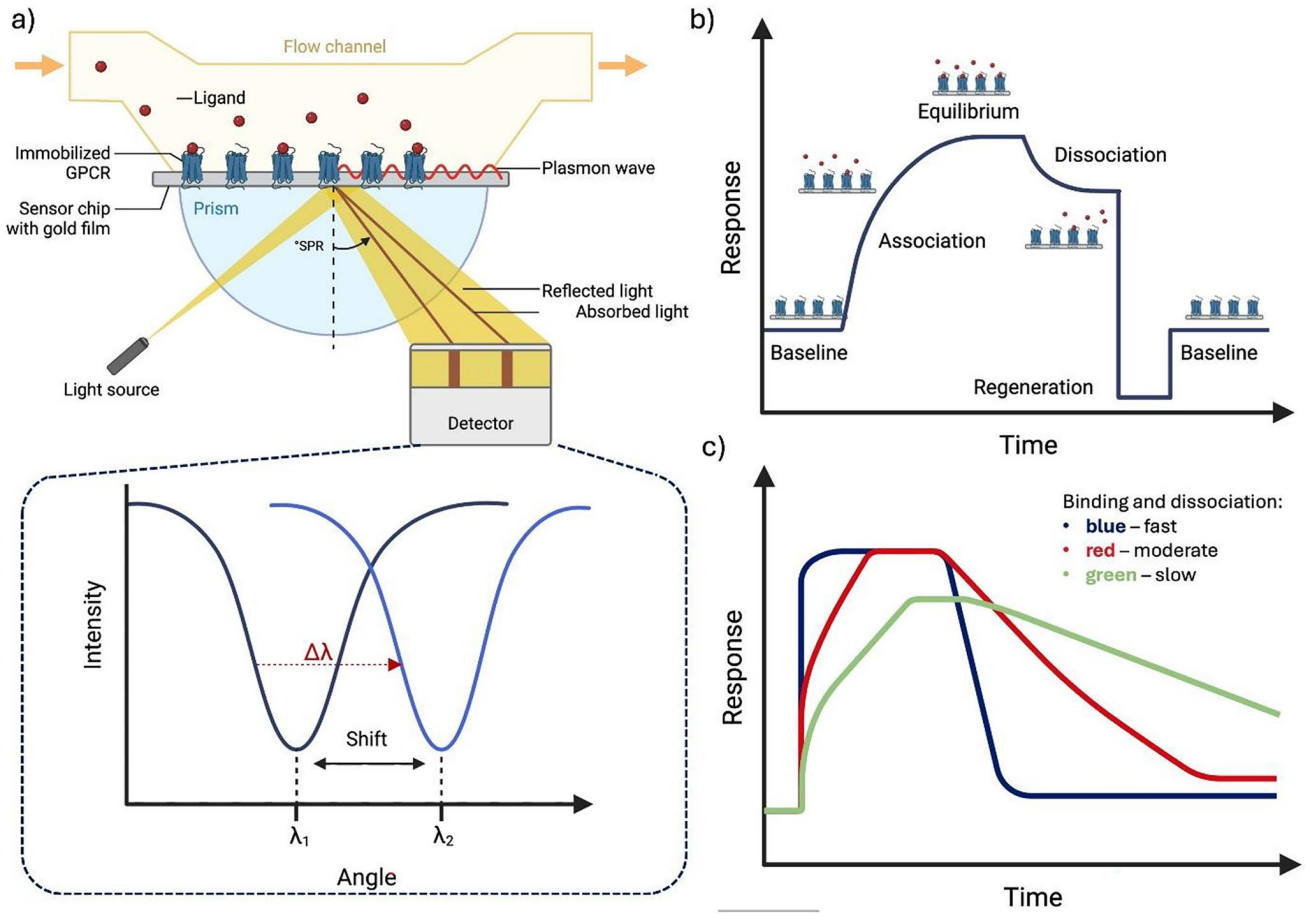
The measurement of ligand RT by SPR [80]. **a**) In SPR, receptors are immobilized on the surface of a sensor chip coated with a thin gold film. Light is directed through a prism surface, where it undergoes total internal reflection, generating an evanescent wave that excites surface plasmons. This interaction produces a characteristic reflection angle (λ_1_), which is recorded by a detector. During the experiment, a ligand is introduced into the flow channel, allowing it to interact with the immobilized receptors. Ligand binding induces a local change in the refractive index, resulting in a measurable shift in the SPR angle from λ_1_ to λ_2_ (Δλ). **b**) Key phases of a SPR experiment. Initially, a baseline signal is established, representing the reflection from the gold film with the immobilized receptors in the absence of ligand binding. Upon ligand injection, the association phase begins, during which ligand molecules interact with the receptors. The signal changes as binding progresses, eventually reaching equilibrium, where the rate of association equals the rate of dissociation. Subsequently, the dissociation phase is triggered by replacing the ligand-containing solution with a buffer, leading to a return of the signal to its initial state as the ligand unbinds from the receptor. Finally, the system undergoes regeneration, restoring the sensor surface to its initial state and returning the signal to the baseline. **c**) The graph presents a comparative analysis of SPR sensorgrams for three examples of binding kinetics according to the rate of association and dissociation

A method that has gained increasing popularity in recent years is surface plasmon resonance (SPR). This label-free, real-time technique involves the use of an SPR sensor chip, where a thin gold layer is deposited onto glass, providing a surface for immobilizing the protein of interest. During the experiment, a polarized light beam passes through a prism and the glass substrate, reflects off the gold layer, and is detected by a photodetector. Binding events are monitored as changes in the intensity of reflected light, either as a function of angle (at a fixed wavelength) or wavelength (at a fixed angle). These intensity changes are sensitive to variations in the refractive index of the material immediately adjacent to the gold layer, in this case, immobilized receptors and ligands from the solution flowing over the chip. Typically, the resulting sensogram (a plot of signal versus time) reveals three phases: association, dissociation, and regeneration of the system to its baseline, the latter being achieved by exposing the receptors to a buffer (it disrupts ligand-receptor interactions and restores the baseline signal, Fig. 3). SPR data provides not only concentration-dependent information but also insights into the kinetics of the process [79]. Two main experimental formats are commonly employed. In the multi-cycle kinetics (MCK) approach, SPR curves are generated for buffer alone and multiple concentrations of the ligand. In contrast, the single-cycle kinetics (SCK) approach, which offers shorter analysis times, involves injecting increasing concentrations of the ligand in a single run, resulting in multiple association phases followed by a single dissociation phase.

Microscale thermophoresis (MST) is a biophysical technique that quantifies biomolecular interactions by measuring ligand-induced changes in thermophoretic mobility within a temperature gradient. This method employs infrared laser heating and fluorescence detection, allowing for sensitive measurements of alterations in molecular properties such as size, charge, conformation, and hydration. These changes reflect binding events and allow for precise determination of binding affinities under near-physiological conditions [81]. MST has been successfully applied to investigate ligand binding kinetics for a variety of biomolecular systems, including membrane proteins. For example it has been used to investigate glutamate binding to ionotropic glutamate receptors iGlu2 and iGlu6, as well as the interaction of small-molecule inhibitors with p38 MAP kinase [82]. Notably, Seidel et al. demonstrated the utility of MST in studying interactions with the neurotensin receptor B (NTS1B) and the adenosine A_2A_ receptor, highlighting its potential for probing GPCR-ligand dynamics [83].

Furthermore, NMR-based methodologies present additional avenues for elucidating ligand-receptor binding kinetics. Advanced techniques such as chemical-shift titration, R₂ relaxation dispersion, and ZZ-exchange experiments enable quantitative characterization of binding dynamics, providing high-resolution insights into the kinetic parameters and conformational exchange processes underlying molecular recognition [84, 85].

### Modeling methods

In addition to experimental studies, the kinetics of ligand binding and compound RT are frequently explored through computational approaches. Among these methods, molecular dynamics (MD) simulations serve as the core of in silico strategies used to assess compound RT and other kinetic parameters. By solving Newtonian equations of motion, MD generates detailed time-resolved trajectories, providing insights into the conformational dynamics, interaction mechanisms, and thermodynamic properties of complex molecular assemblies. Classical MD simulations, while offering atomic-level insights into biomolecular processes, are fundamentally constrained by their limited temporal reach, typically on the order of nanoseconds to microseconds. This duration is insufficient to observe ligand unbinding events for many drug-like molecules, which often exhibit RTs on the scale of minutes to hours. Despite the advent of state-of-the-art computational resources, including high-performance GPUs and specialized hardware such as Anton, Anton 2, and MDGRAPE series, the stochastic nature of rare-event dynamics, coupled with the high free-energy barriers characteristic of ligand unbinding, limits the ability of conventional MD to capture these processes. These temporal constraints underscore the need for enhanced sampling techniques and algorithmic innovations to extend the effective simulation timescales, enabling a more comprehensive characterization of unbinding kinetics and associated free-energy landscapes. In this review, we will focus on the main group of MD approaches used in the compound assessment in terms of their kinetics. While classical MD generally cannot capture ligand unbinding events due to their rarity and long timescales, there are cases where dissociation has been observed, particularly for low-affinity compounds [86]. Classical MD has also been employed to assess the overall behavior of compounds within the binding pocket, with attempts to monitor changes in ligand-protein interaction networks that may influence binding duration [87]. Nevertheless, classical MD is not widely applied to kinetic studies and ligand unbinding, as capturing such rare events typically requires the use of enhanced sampling techniques.

**Fig. 4:**
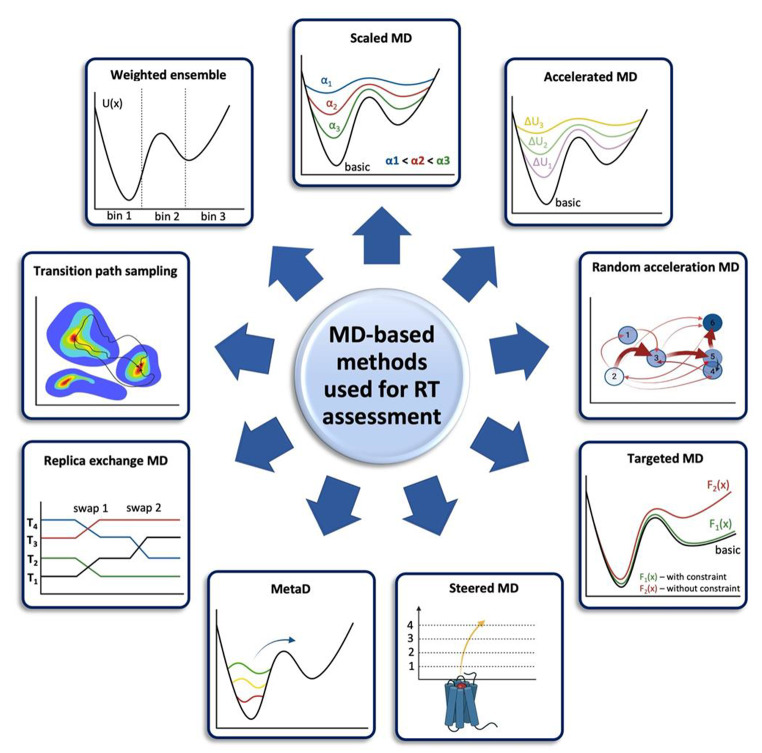
Different types of MD-based methods used for RT prediction [172]. Different types of MD-based methods used for RT prediction: scaled MD: applies a scaling factor α to energy terms; accelerated MD: a biasing potential ΔU is added to smooth the free energy landscape; random acceleration MD: applies randomly oriented forces to promote transitions between energy minima; targeted MD: applies a biasing potential/constraint F to guide the system towards the target state; steered MD: an external force is applied to pull ligand from the binding pocket; MetaD: introduces history-dependent bias potentials to selected collective variables; replica exchange MD: multiple simulations at different temperatures are run with the periodic swap of configuration; transition path sampling: a collection of transition trajectories between two states is produced

One approach that modifies classical MD simulations is **scaled MD (sMD)**. This method introduces a scaling factor (ranging between 0 and 1; Fig. 4) to reduce the potential energy surface (PES), facilitating transitions between metastable states and increasing the likelihood of observing rare events [88–90]. For instance, sMD has been applied to a series of Hsp90 inhibitors with affinity differences spanning three orders of magnitude. Using sMD, RTs of structurally similar compounds were successfully ranked [91], and unbinding times of ligands of human d-amino acid oxidase were determined [92]. A key limitation of sMD lies in the selection of the scaling factor, which is typically determined through empirical approximation, rather than via the well-defined theoretical basis [93–95]. Furthermore, overly aggressive scaling can lead to unrealistic ligand trajectories [93]. Another drawback of sMD is the need for additional constraints to maintain the protein’s secondary structure while ensuring sufficient conformational flexibility at the binding site, complicating the setup process [94].

DebFrank’s group proposed an alternative to accelerate ligand dissociation during simulations by selectively scaling interactions. Instead of scaling the entire PES, this approach selectively scales specific non-bonded contacts, indirectly modulating protein-ligand interactions [95, 96]. This strategy eliminates the need for additional constraints to prevent protein unfolding. Using Kramers-based rate extrapolation and free energy extrapolation, the** selective scaling MD (ssMD)** approach demonstrated utility in predicting RTs and changes in the system’s Gibbs free energy, with results showing good agreement with experimental data [95]. A further advancement of ssMD involved the adoption of machine learning techniques to improve accuracy of RT predictions [96]. A similar concept of combining MD simulations with machine learning was also implemented by Du Wang in the local-scaled MD, where the selected region of contacts around the ligand-protein interface is adjusted [97].

Another technique used to study rare events, such as ligand dissociation, is **accelerated MD (aMD)**. In aMD, the time required to cross energy barriers is reduced by applying a biasing potential to a selected collective variable (e.g., potential energy or distance between key atoms). This leads to the flattening of the PES in certain regions, facilitating energy barrier crossing [98–100]. Although aMD has been successfully applied to kinetic studies [101], it is increasingly being replaced by **Gaussian accelerated molecular dynamics (GaMD)**, in which a harmonic biasing potential is used to smoothen PES. Further development of GaMD resulted in the introduction of LiGaMD, a variant in which the potential energy of ligand non-bonded interactions is selectively boosted using the GaMD framework [102]. For instance, LiGaMD has successfully modeled benzamidine binding to trypsin [103]. Subsequent refinements resulted in LiGaMD2 [104] and LiGaMD3 [105]. In LiGaMD2, the boost potential is applied to both the ligand and surrounding residues in the protein pocket, whereas LiGaMD3 uses triple boosts on three individual energy terms: non-bonded interaction energy of the substrate, remaining non-bonded potential energy of the system, and thebonded potential energy of the entire system. Another modification of GaMD, known as PPI-GaMD, has been developed to study protein-protein interaction kinetics [106]. A comprehensive review of GaMD and its variants is provided in Wang, 2021 [107].

While sMD achieves acceleration by scaling the potential energy function derived from the force field (and thereby lowering energy barriers which facilitates ligand dissociation), **random acceleration molecular dynamics (RAMD)** employs a different strategy. RAMD applies an additional stochastic force to the ligand in random direction which pulls it out of the binding pocket, and therefore enables exploration of multiple dissociation pathways. Whereas sMD modifies the energy landscape by smoothing the PES, RAMD adds external perturbations and is particularly effective in estimating dissociation rates (typically RAMD can induce ligand dissociation within simulation timescales of tens to hundreds of nanoseconds). As an example of its application, RAMD has been used to calculate RTs for inhibitors of heat shock protein 90α (Hsp90) [108], incorporating machine learning techniques and interaction fingerprints to refine the final predictions [109]. It has also been applied to study the dissociation mechanisms of B-RAF inhibitors [110], as well as benzene and indole in T4 lysozyme mutant complexes [111], and inhibitors targeting focal adhesion kinase and proline-rich tyrosine kinase 2 [112].

Another method that applies external force in the system is **targeted MD (TMD)** [113]. It identifies a specific target state for the system, and the simulation is designed to facilitate the transition from the initial state to that target. The system’s progress is typically characterized by the root mean square deviation (RMSD) from the target structure. To promote convergence toward the desired state, a biasing force or harmonic restraint is applied, aimed at reducing the RMSD over the course of the simulation and guiding the system along a predefined pathway. To preserve the integrity of the natural transition pathways and minimize potential distortions, TMD is often coupled with other enhanced sampling methods. For instance, Wolf et al. employed TMD to study molecular mechanisms of ligand unbinding from Hsp90 [114]. In a subsequent study, the authors applied dissipation-corrected targeted molecular dynamics (dcTMD), in which a pulling force is applied along the reaction coordinate using a moving distance constraint. This approach, combined with temperature-boosted Langevin simulations, was used to study the trypsin–benzamidine and Hsp90 complexes, successfully capturing ligand dissociation events [115].

Another technique that employs external forces for the controlled manipulation of molecular systems in ligand kinetics examination is **steered MD**. In this method, a pulling force is applied to selected atoms within the system. Unlike TMD, where the bias is guided by the RMSD to a target structure, steered MD directly controls the magnitude of the applied force or velocity of pulling. It constitutes the basis for the division of steered MD into two groups, which use constant-force pulling or constant-velocity pulling, respectively. Example applications of steered MD include the investigation of unbinding mechanisms of cyclin-dependent kinase 5 [116] and bovine β-lactoglobulin/Schistosoma japonicum glutathione S-transferase tyrosine 7 [117]. In the context of GPCRs, compounds such as tetrahydrocannabinol and anandamide which were pulled out of the binding pocket of cannabinoid CB_1_ receptor with the constant velocity [118]. An analogous approach was used to the extended set of receptors and their ligands: rhodopsin, cannabinoid CB_1_, sphingosine-1-phosphate S1P1, and lysophosphatidic LPA1 [119]. Furthermore, adenosine receptor A_2A_ modulators, antagonist ZMA241385 and agonist NECA, were examined using a combination of steered MD and the Bell-Evans model in terms of their unbinding pathways and kinetic parameters estimation [120].

Apart from scaling PES or applying external forces, **metadynamics (MetaD)** represents another enhanced sampling method designed to explore rare events [121]. In MetaD, the bias is applied within a low-dimensional space defined by collective variables. This biasing potential is introduced in a history-dependent manner, with contributions being added at regular intervals throughout the simulation. As the simulation progresses, these contributions accumulate, creating a dynamic and evolving potential landscape that reflects the progression of the system’s exploration. In its well-tempered variant (WT-MD), the exploration of the energy landscape is refined through incremental adjustments of the biasing potential. This approach ensures the systematic exploration of new regions while simultaneously preventing the overfilling of energy wells. As a result, a balance is achieved between exploration and exploitation, enhancing both the efficiency and accuracy of the simulation [122].

MetaD has been extensively employed to study ligand binding in diverse systems. Notable examples include studies on the p38 MAP kinase Type II [123], and its WT-MD variant: hypoxia-inducible factor 2α [124], inhibitors of cyclin-dependent kinase 8 [125], 5-FU and uracil binding to the yCD enzyme [126], and antagonists of the M_3_ muscarinic receptor [127]. WT-MD has also been utilized for virtual screening of compounds targeting the ATP site in the pseudokinase domain of JAK2 kinase [128], as well as for investigating fentanyl derivatives and opioids binding to the µ-opioid receptor [129] and ligands interacting with the threonine-tyrosine kinase receptor [130].

MetaD faces challenges when studying rapid ligand dissociation events, as unbound ligands may adopt numerous solvent-exposed conformations after detaching from their binding sites, complicating sampling efficiency and data interpretation. Funnel metadynamics (FM) addresses this issue by employing a narrowing funnel-shaped restraining potential that guides the ligand along its dissociation pathway while limiting irrelevant solvent exploration. This enhances the study of ligand kinetics, RTs, and association/dissociation rates. Proper placement of the funnel and selection of appropriate collective variables are crucial for the method’s success [131]. Applications of FM to examine the ligand unbinding from its target include studying GABA unbindingfrom the insect RDL receptor [132], peptide interactions with neuropeptide Y receptors, vasopressin V_2_ receptor, and oxytocin receptor [133], as well as studies on inhibitors targeting methionine aminopeptidase-II [134].

A notable advancement within the MetaD framework is the development of infrequent metadynamics (iMetaD), which applies bias infrequently and terminates trajectories once predefined criteria are met, focusing computational resources on significant transitions. The resulting first-passage times are then rescaled using an acceleration factor, enabling the extraction of reliable kinetic properties with reduced computational overhead [135, 136].

Enhanced sampling is also obtained in **replica exchange molecular dynamics (REMD)**. It employs multiple replicas of a system, each running in parallel at a different temperature. These replicas periodically exchange configurations, facilitating transitions between high- and low-energy states and allowing for the exploration of states that might be inaccessible in a standard MD runs due to high energy barriers. Although REMD is not inherently designed for kinetic analysis, it has been applied in this context. For example, Stelzl and Hummer [137] used REMD to gaininsights into the kinetics of folding for both alanine dipeptide and the neomycin RNA riboswitch. Additionally, Shinobu and co-workers combinedKliknij lub naciśnij tutaj, aby wprowadzić tekst. combined the generalized replica exchange with solute tempering (gREST) with the replica-exchange umbrella sampling (REUS) in kinase-inhibitor binding simulations, and successfully observed multiple ligand binding/unbinding events [138]. Nevertheless, despite several notable examples of successful REMD applications, extracting precise kinetic information from such simulations necessitates careful consideration of various factors, particularly exchange frequency between replicas. Frequent exchanges may induce unphysical transitions and distort kinetic rates, whereas infrequent exchanges can hinder effective sampling of the energy landscape, leading to incomplete or biased results.

Another group of methods used in ligand kinetics examination is **transition path sampling (TPS)**. This technique generates an ensemble of reactive trajectories connecting bound and unbound states by performing a Monte Carlo random walk-in trajectory space. Unlike energy-biased approaches, TPS does not require predefined reaction coordinates or collective variables. By selectively sampling only the trajectories that successfully navigate the energy barrier, TPS offers a statistically reliable representation of transition pathways, facilitating the calculation of important kinetic parameters like ligand RT. An advancement of this technique, transition interface sampling (TIS), uses a sampling strategy that focuses on the transition interface and focuses on the critical regions of the energy landscape, capturing information from the neighborhoods of initial and final states.

TPS strategies are often combined with the Markov state modeling (MSM) [139, 140] as a framework that transforms the simulation data generated through TPS into a probabilistic roadmap for studying biomolecular interactions. Markovian transitions refer to state-to-state changes in a stochastic system governed by the Markov property, wherein the probability of transitioning to a future state depends exclusively on the current state, independent of the system’s prior history. MSMs effectively capture the dynamics of complex molecular systems by discretizing conformational space into metastable states. MSMs utilize ensembles of shorter trajectories to reconstruct the complete kinetic landscape of ligand binding and unbinding, thereby enhancing the understanding of rare dissociation events. MSMs is particularly well-suited for capturing the heterogeneity of unbinding pathways, revealing multiple kinetically relevant routes and intermediate states that influence overall RT. This multi-pathway perspective is crucial for understanding ligands whose unbinding kinetics are affected by conformational changes or solvent-mediated transitions. Moreover, MSMs enable state-specific rate calculations, helping researchers identify which intermediate states significantly impact observed kinetics.

Another powerful technique for studying ligand kinetics is the **weighted ensemble (WE) **method. It is based on evolving multiple copies of a system (walkers) and dynamically adjusting their weights to ensure unbiased sampling of the transition path between ligand-bound and unbound states. A key component of WE is the use of bins to discretize the reaction coordinate or the relevant progress variable (e.g., the distance between the ligand and the receptor). The bins are used to divide the phase space into regions that correspond to different stages of the reaction (bound/unbound states in the considered case). Thanks to the binning approach, a focus of computational resources is placed on the most relevant transitions. Examples of its successful applications include the exploration of the escape pathways of a small molecule from T4 lysozyme [141]. Meanwhile, *Lotz and Dickson* [142] introduced Wepy, a versatile software framework designed for efficiently simulating rare events through a weighted ensemble resampling method. Complementarily, Ahn et al. focused on the β-cyclodextrin ligands [143].

The most popular MD-based methods applied in kinetic studies are illustrated in Fig. 4. Their technical details are summarized in Table 1, while the advantages and limitations of each method in the context of RT assessment are discussed in Table 2.

**Table 1:** Summary of the most popular enhanced sampling techniques used in MD-based studies of compound RT

MD variant	Technical principle	
Scaled MD (sMD)	Applies a scaling factor to the potential energy function or specific interaction terms to lower energy barriers.	
Accelerated MD (aMD)	Adds a boost potential to flatten energy barriers.	
Random acceleration MD (RAMD)	Applies a random force to particles in the system, continuously adjusted to avoid bias in a single direction.	
Targeted MD (TMD)	Guides a system toward a specific target state by applying a harmonic biasing potential, often aimed at reducing RMSD between the system and target structure.	
Steered MD	Applies an external directional force to pull a ligand away from the binding site.	
Metadynamics (MetaD)	Applies a time-dependent biasing potential to the system along selected collective variables in such a way that the system is pushed away from local energy minima.	
Replica exchange molecular dynamics (REMD)	Runs multiple replicas of the system in parallel at different temperatures (or other thermodynamic conditions), and periodically exchanges configurations between these replicas.	
Transition path sampling (TPS)	Generates a collection of spontaneous transition paths between two states (bound/unbound), typically with the use of Monte Carlo or Markov chain sampling.	

**Table 2:** Advantages and disadvantages of different MD methods in the context of RT assessment

MD variant	Strengths	Limitations
Scaled MD (sMD)	-useful for relative ranking (widely used for qualitative RT ranking)	-difficult quantitative determination of koff -can be artificial due to high bias
Accelerated MD (aMD)	-no specific biasing direction or coordinate needed -captures rare transitions without requiring prior knowledge	-difficult koff estimation without extensive reweighting (which is often inaccurate) - biased potential complicates direct kinetic interpretation
Random acceleration MD (RAMD)	-relatively low computational cost -efficiently maps multiple ligand egress routes -minimal prior knowledge needed -good choice for cases in which the binding/unbinding mechanism is not well understood	- absolute k_off_ values not directly obtained but can be estimated from the unbinding time distribution across replicas - typically requires many repeated runs to build a statistically significant picture - ignores slow conformational rearrangements due to the artificially fast unbinding
Targeted MD (TMD)	- transition path from TMD can seed umbrella sampling or metadynamics along a physically reasonable reaction coordinate	- -requires defining the target structure of the system - -high system perturbation (artificial trajectories) - suppresses natural fluctuations and conformational sampling critical for accurate kinetics
Steered MD	- intuitive and easy to set up - good for comparing relative unbinding strengths -when used in conjunction with Jarzynski’s equality or work-based methods, it can effectively approximate the free energy profiles along the unbinding pathway.	-limited conformational exploration - pulling speed and direction can alter results significantly - artifacts at high pulling velocities (e.g., unfolding or denaturation).
Metadynamics (MetaD)	-enables quantitative estimation of k_off_ - can capture transition states and intermediate conformations along the dissociation pathway, providing more detailed information about the mechanism of ligand-receptor unbinding -allows the use of different biasing potentials, which can be tailored to target specific degrees of freedom	- requires a well-chosen CV - can overfill free energy wells if not carefully tuned -difficult and error-prone reweighting for kinetics
Replica exchange molecular dynamics (REMD)	- realistically samples protein flexibility	- requires many replicas, making it computationally expensive - does not specifically drive ligand unbinding (improves sampling globally, it might not efficiently explore slow dissociation events) - high computational cost
Transition path s ampling (TPS)	-does not require modification of the energy landscape -suitable for systems when unbinding pathways are not known -provides realistic, unbiased, unbinding pathways -enables determination of k_off_	-complex setup and steep learning curve -success depends on having precisely defined initial and final states
Weighted ensemble (WE)	-enables determination of k_off_ -unbiased (preserves realistic trajectories and forces) -suitable for high-performance computing environments (inherently parallel method)	-requires specification of progress coordinates and binning schemes -complex setup and non-trivial learning curve

The accurate prediction of ligand RT via MD-based methods is critically dependent on both enhanced sampling strategies and key technical parameters within the MD setup. Crucial factors include the choice of force fields, ligand parameterization, and the methods used to compute partial charges. Different force fields, such as AMBER, CHARMM, and OPLS, employ distinct parameterization schemes that affect intermolecular interactions and the free energy barriers governing dissociation kinetics [144, 145]. Ligand parameterization is particularly important, as imprecise representations can lead to unphysical geometries and skewed binding kinetics. Moreover, the assignment of partial atomic charges significantly influences long-range electrostatic interactions, which can stabilize or destabilize intermediate states. Common methods for charge assignment range from rapid semi-empirical approaches to more accurate RESP and DFT-derived [146–149]. Given the sensitivity of RT predictions to these variables, it is vital to meticulously report parameter choices and consider multiple models in the analysis.

### Molecular insights into RT assessment– selected case studies

Understanding the determinants of ligand RT necessitates a detailed investigation into the molecular mechanisms governing ligand dissociation from its target and the barriers that impede unbinding, thereby prolonging RT. A key hypothesis suggests that prolonged RT arises from persistent interactions with critical binding site residues, which modulate the ligand dissociation kinetics.

In MD simulations of the β2-AR bound to an agonist, antagonist, and inverse agonist (denoted P0G, JTZ, and JRZ, respectively), distinct differences emerged between the receptor in its G protein-bound and unbound states. Notably, in the absence of a G protein, a greater number of low-energy conformational states were observed, suggesting that G protein coupling stabilizes the active conformation of the receptor. A key dissociation pathway was identified, characterized by the displacement of the 6^th^ transmembrane helix TM6 away from its active conformation while moving toward TM3 and TM5. Furthermore, ligand dissociation simulations of the receptor in the G protein-unbound state revealed the formation of a stabilizing salt bridge between Arg3.50 and Glu6.30, alongside alterations in hydrophobic interaction networks involving TM3, TM5, and TM6. These structural features are hypothesized to contribute to extended ligand RT by increasing energetic barriers to dissociation [150].

The molecular basis of ligand RT has been extensively investigated across various protein targets, including Hsp90, opioid receptors, kinases, and MDM2. In the case of Hsp90, RAMD simulations conducted on a set of 94 ligands enabled their classification into clusters based on RT. Ligands exhibiting slow dissociation and prolonged RT were characterized by extensive hydrophobic interactions with residues lining a hydrophobic pocket adjacent to α-helix 3 and its entrance. A key determinant of extended RT was the presence of a carbonyl moiety in the ligand, which formed a stabilizing interaction resembling a halogen bond, particularly with residues N51–F138. Notably, the anticipated hydrogen bond with N51 was found to be non-essential. Additionally, ligands with the longest RT consistently exhibited hydrogen bonding with Y139. Furthermore, alicyclic and methoxy-substituted ligands preferentially occupied the hydrophobic binding pocket, correlating with prolonged RT. Similar observations were reported by Schuetz, who emphasized the role of binding site residues in modulating ligand unbinding kinetics. The presence of specific functional groups within the hydrophobic pocket was found to impede dissociation by requiring the disruption of multiple stabilizing interactions [91].

In the case of fentanyl derivatives, structural modifications of substituents were found to modulate RT, with variations in dissociation kinetics correlating with the binding energy of the substituents at the µ-opioid receptor. Notably, interactions with H297 were identified as the primary determinant of RT, while the strongest ligand-receptor interaction, observed with D147, exerted minimal influence on RT [129]. The significance of ligand-protein interactions in modulating RT has been corroborated by multiple studies employing enhanced sampling techniques, including MetaD [151], sMD [95], and free-energy perturbation methods [94].

In the case of Vasopressin V_2_ receptor antagonists, structure-kinetics relationship analysis identified factors contributing to prolonged RT, such as the presence of a bulky substituted aromatic system in the benzamide moiety and the substitution of an electron-withdrawing group at the meta position of the phenoxy group [152].

Furthermore, for the cell division protein kinase 8 (CDK8), ureido and carbamoyl functional groups were found to prolong RT by engaging in hydrogen bonding with Lys52, thereby delaying ligand dissociation from the active site [125]. For focal adhesion kinase inhibitors, hydrophobic interactions with M499, D564, and L567 were identified as critical determinants of RT, with their absence leading to rapid dissociation. Additionally, interactions with L567 were shown to stabilize the helical conformation of the DFG motif, thereby increasing both RT and ligand affinity. Site-directed mutagenesis (FAK L567A) confirmed the importance of this interaction in RT modulation. Comparative studies of FAK and PYK2 further demonstrated that binding selectivity between these kinases arises primarily from differences in dissociation kinetics rather than initial binding affinities [112].

The importance of particular ligand-protein contacts in prolonging RT can be examined experimentally e.g., via the site-directed mutagenesis. Mutating residues in the binding pocket can weaken critical ligand-protein contacts and result in faster ligand dissociation or introduction of new interactions which can stabilize the ligand-protein complex, leading to the prolonged RT. Receptor mutations can also influence RT by modulating the stability of the transition states, blocking or creating exit pathways, or affecting the solvation effects [153].

### Conformational barriers and their role in ligand RT

Another critical factor influencing ligand RT is the presence of conformational transitions that require overcoming substantial energy barriers. Studies on Hsp90 ligands has demonstrated that polar interactions and steric hindrances during dissociation can significantly extend RT. In particular, interactions with α-helix 3 were identified as a key determinant, as ligand dissociation in such cases necessitated substantial conformational rearrangements of both the ligand and the protein. This leads to the formation of an energetic “cage” (or an energy well), which creates a kinetic barrier that effectively delays dissociation [108].

Investigations of benzene exit pathways from T4 lysozyme further revealed multiple ligand entry and exit routes to and from the active site. Notably, one of these pathways was found to be unidirectional due to the conformational changes in residues VAL149 and MET102 upon benzene binding. These structural rearrangements effectively closed off the dissociation pathway, thereby preventing ligand unbinding [154].

These findings highlight the significance of conformationally gated dissociation pathways in determining ligand RT, emphasizing the interplay between steric constraints, protein flexibility, and energetic barriers in modulating ligand-protein interactions.

### Conformational dynamics of proteins and their influence on ligand RT

Conformational changes within the protein structure are increasingly recognized as critical determinants of ligand RT. One such mechanism involves the presence of an active site “lid”, a structural feature that modulates both ligand binding and dissociation. The “lid” formation can result in various effects, such as the presence of additional ligand-protein contacts between ligand and amino acids out of which the “lid” is composed, which further stabilize the compound in the binding pocket. Additionally, the “lid” residues can create a physical barrier, which hinders the ligand’s ability to freely dissociate from the receptor [155, 156].

MD simulations of human D-amino acid oxidase have revealed a flexible loop that acts as a gating mechanism for the ligand-binding pocket. Two distinct dissociation pathways were identified: in the first, an open lid conformation allows water molecules to enter the binding pocket, thereby facilitating the disruption of ligand-protein interactions and accelerating dissociation. In the second pathway, a more rigid lid conformation restricts pocket accessibility, necessitating direct ligand-protein bond cleavage for dissociation. Computational analyses indicate that ligands dissociating via the second pathway exhibit faster unbinding, whereas those following the first mechanism show prolonged RT, suggesting that the lid structure may serve as a kinetic barrier to unbinding.

A similar phenomenon was observed in the dissociation of 5-fluorouracil (5-FU) from yeast cytosine deaminase. The slower dissociation of 5-FU, relative to uracil, was attributed to the presence of multiple metastable bound states. Structural analysis further identified a C-terminal lid, with fluctuations in the 11–117 loop and C-terminal residues playing a crucial role in facilitating ligand dissociation [126]. Comparable gating mechanisms were reported for the ionotropic GABA receptor, where fluctuations in the C-loop conformation, quantified via changes in loop angle and RMSD of key residues, were found to be essential for GABA unbinding [132]. Another example is constituted by enoyl-ACP reductase (saFabI) inhibitors, for which the closure of the substrate-binding loop stabilized by favorable interactions within the binding site, resulting in the extended RT [155].

Noteworthy findings from 5-HT_2B_ receptor crystal structures highlighted the critical role of residue Leu209 in the ECL2. Ligands interacting with this residue not only exhibit enhanced RT but also promote time-dependent β-arrestin recruitment. Furthermore, these ligand-lid interactions influence both TM7 and ECL2, stabilizing the ligand-receptor complex, as clearly demonstrated by the receptor’s crystallographic structure [156].

The extended RT of LSD at the 5-HT_2B_ receptor also appears to be influenced by a structural “lid” mechanism formed by ECL2. In the LSD-bound 5-HT_2B_R structure, residues 207–214 of ECL2 create a lid over the ligand, likely obstructing its dissociation and thereby prolonging its RT. Structural comparisons with the 5-HT_2B_R/ERG complex obtained experimentally reveal a more open conformation in the latter, suggesting that the degree of EL2 coverage correlates with ligand dissociation kinetics [157].

A similar mechanism has been observed in β_2_-AR activation, where ECL2, specifically F193 in ECL2, interacts with Y308^7.35^ from TM7, forming a lid-like structure that stabilizes the active state. This, along with other studies [3, 24, 158–161], suggests that ECL2 may serve as a key regulatory element across different GPCRs, modulating ligand binding kinetics and prolonging receptor signaling through steric hindrance and conformational stabilization [162].

### Protein flexibility as a modulator of ligand kinetics

While protein flexibility has been proposed as a key factor governing ligand dissociation, the extent of its influence remains under debate. Structural adaptations observed during benzene binding to T4 lysozyme suggest that unbinding, unlike binding, requires enhanced conformational flexibility. During dissociation, benzene transiently occupied a nearby hydrophobic cavity, inducing a conformational transition in the protein. This higher-energy intermediate state exposed a more polar surface, ultimately facilitating full ligand dissociation. Subsequent simulations confirmed that in the T4 lysozyme-benzene system, protein flexibility can drive opening or closure of binding pockets and even promote the formation of novel binding sites [104].

Entropy-driven binding has also been implicated in modulating RT, particularly for N-terminal Hsp90 inhibitors. Studies indicate that ligand binding in this system is predominantly driven by entropic factors, with conformational flexibility in the bound state playing a crucial role. Ligands stabilized through entropic contributions exhibit both slow binding and slow dissociation kinetics, resulting in prolonged RT. This suggests that the formation of more dynamically adaptable active complexes can extend ligand RT [163]. Similar conclusions were reached by Callea et al. using adaptive MD (variation of MetaD), further reinforcing the link between protein conformational plasticity and ligand kinetic stability [124].

These findings highlight the intricate relationship between protein flexibility, binding site gating mechanisms, and the energetic landscape of ligand unbinding. A deeper understanding of these factors could prove instrumental in the rational design of inhibitors with optimized kinetic properties.

### The solvent influence on compound RT

Significant findings also emphasize the role of water in the dissociation process. These conclusions challenge the notion that protein flexibility is necessary, instead supporting the importance of conformational stability. The addition of phenyl and naphthyl groups to the urea nitrogen resulted in a 100- to 1000-fold decrease in *k*_*off*_ (and consequently, a substantial increase in RT). It has been suggested that this effect is attributed not only to the enhanced stabilization of ligand-protein interactions but also to the filling of the active site with hydrophobic groups, which restrict water accessibility. Reduced water penetration minimizes disruptions in ligand-protein interactions [123]. A similar observation was reported by Cavalli et al. [164].

In the case of p38α MAPK inhibitors, key factors determining differences in RT were identified as protein conformational stability and solvent exposure. The impact of water—facilitating ligand dissociation and its resolvation in the “extra receptor” environment—was found to be more energetically favorable and kinetically accessible for ligands with short RT. These findings contrast with previous observations suggesting that that emphasized the importance of protein flexibility. Short-RT inhibitors were characterized by a lower percentage of buried surface area. It is hypothesized that this effect is driven by reduced protein flexibility (favoring a more stable conformation) and the associated decrease in water influence on the bound ligand [165].

A similar role of water in ligand dissociation was observed by Ansari et al. in the case of benzamidine unbinding from trypsin. The dissociation mechanism begins with the entry of an additional water molecule, which induces a new conformational state in the system. In the case of rapid dissociation, water weakens the ligand–W1 interaction, leading to the formation of a water–Asp189 hydrogen bond, ultimately resulting in LR complex disassembly. In the slower dissociation mechanism, water forms a bridging interaction between the ligand and Asp189, leading to dissociation, but at a slower rate (with an extended RT) [166].

In the case of ligand dissociation from the CRF1 receptor, a low degree of ligand solvation during unbinding was observed to prolong RT. Key residues contributing to this effect were also identified. Additionally, hydrophobic shielding of hydrogen bonds was found to have a positive correlation with the prolonged RT [167].

### Interaction of GPCR ligands with the secondary binding pocket with reference to compound RT

The RT of GPCRs is also influenced by ligand interactions with the secondary binding pocket (SBP). Engagement with the SBP establishes additional contacts that stabilize the ligand within the target and extend its occupancy. Moreover, SBP plays an important role in properly orienting a compound during both association and dissociation events. An effect of prolonged RT for compounds occupying SBP or being placed in the proximity of SBP was observed for example for the histamine H_1_ receptor, where a significant increase in RT was noted when shifting from desloratadine to rupatadine, the latter forming additional interactions with the SBP, contributing to its extended duration of ligand-protein complex [167]. Similarly, salmeterol, a bitopic β_2_AR ligand, exhibited over 5-fold longer RT compared to salbutamol and epinephrine, which bind exclusively to the orthosteric site [168].

### Compound lipophilicity

Compound RT can also be influenced by its general physicochemical properties. One of the crucial features in this context is lipophilicity. Ligands with higher lipophilicity tend to be characterized by increased RT, as their increased hydrophobicity facilitates formation of contacts with the hydrophobic regions of the target, and more stable binding translates to increased RT. The above-mentioned relationship was reported, e.g., for the dopamine D_2_R ligands [169], C-C chemokine receptor type 2 [170], and cannabinoid receptor CB_2_ [171]. However, it is important to maintain a balance in lipophilicity. Excessive lipophilicity may lead to undesirable effects, such as increased off-target binding or elevated local concentrations due to a greater tendency for membrane partitioning [161].

## Conclusions

**Fig. 5:**
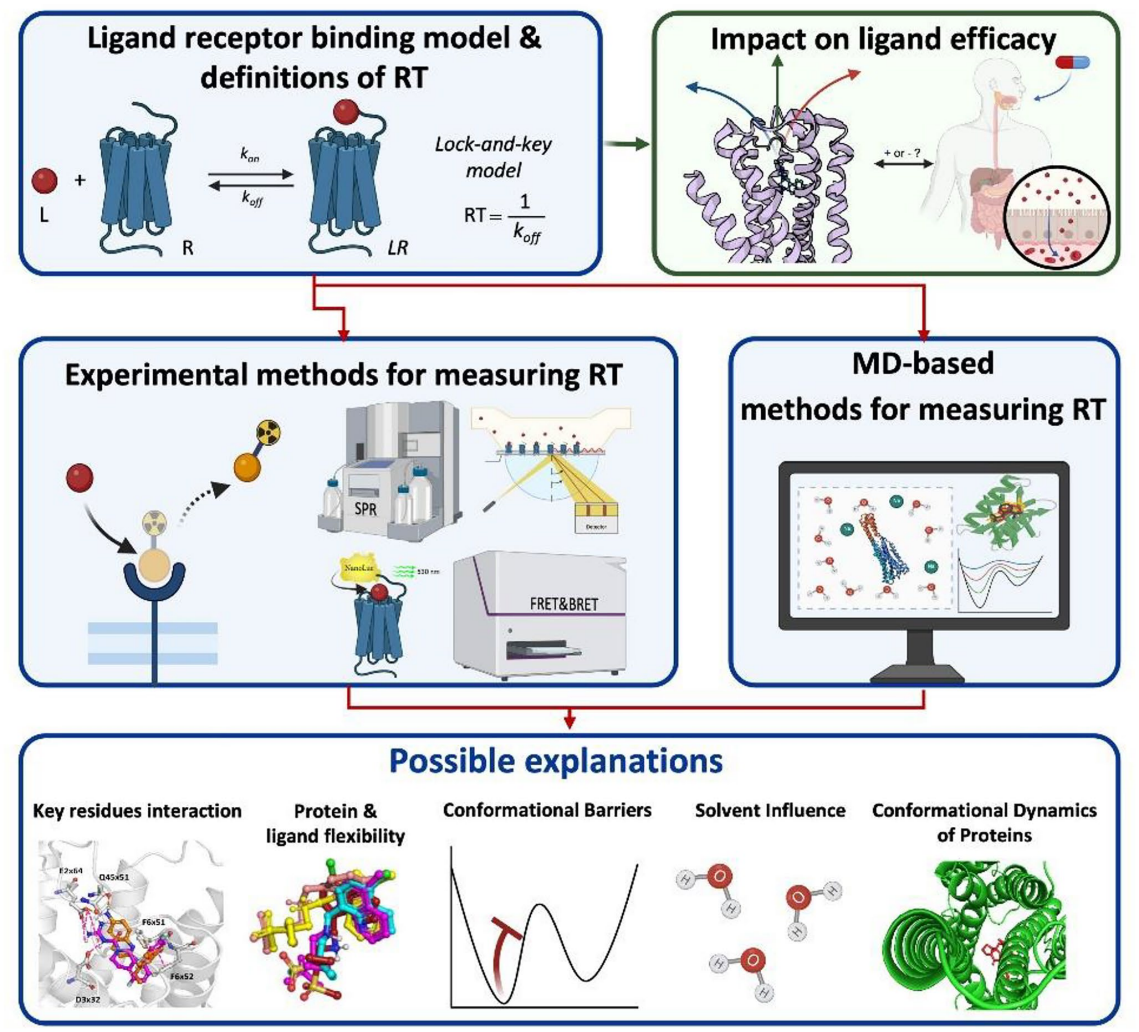
The main findings in computational approaches for identifying molecular determinants of prolonged RT [173]

This review highlights the important role of RT in drug design and discusses its impact on drug efficacy. It summarizes arrange of experimental techniques used for RT determination, including radioligand- and non-radioligand-based methods. Moreover, MD-based protocols for in silico assessment of ligand RT are discussed and the molecular determinants of prolonged RT for selected cases are reviewed (Fig. 5).

Most MD-based research findings on different factors influencing RT focus on the formation of a hypothetical cage within the binding site. This phenomenon can be conceptualized as energetic confinement, wherein the ligand is kinetically trapped due to structural and dynamic constraints imposed by the protein environment. The ligand ligand might be viewed as a “prisoner” trapped within the protein or as a “fly” whose own interactions trigger conformational rearrangements that prevent its escape, similar to an insect ensnared by a carnivorous plant. It is well established that this “cage” is associated with one or more energetic minima, surrounded by high-energy barriers that impede ligand escape. Empirical studies reveal numerous factors contributing to the formation and stabilization of this energetic cage, which, in some cases, manifests as a physical enclosure. One of key contributors is the presence of stabilizing interactions that maintain a ligand in a low-energy state. Another notable effect is the formation of a structural “lid” that restricts ligand egress from the binding site. Additionally, water plays a significant role via reduction of the likelihood of ligand resolvation or weakening of the ligand-protein interactions by disruption of ligand-protein contacts.

Ultimately, this review underscores the importance of integrating experimental and computational methods to deepen our understanding of the RT phenomenon. A comprehensive overview of current experimental and in silico methodologies indicates that the accurate predictions of RT remain a significant challenge, and that the continuous improvement of in silico methods, which can help to bridge the gap between simulations and experimental observations, is needed. Advancing this field holds promise for the rational design of ligands with optimized kinetic profiles, ultimately contributing to the development of more effective therapeutic strategies.

A fundamental yet unresolved question in drug discovery is which molecular features govern the extension or reduction of ligand–receptor RT, and to what extent RT correlates with pharmacological efficacy, including in vivo models. Structural imaging techniques such as X-ray crystallography and, more promisingly, cryo-electron microscopy offer valuable opportunities to elucidate these mechanisms at high resolution. Furthermore, there is a growing need for the development of more reproducible and computationally efficient protocols capable of accurately predicting RT, particularly in the context of high-throughput screening.

## Data Availability

No datasets were generated or analysed during the current study.

## Abbreviations

5-FU: 5-fluorouracil
β_2_-AR: β2 adrenergic receptor
aMD: Accelerated molecular dynamics
ADME: Absorption, distribution, metabolism, and excretion
AT_1_R: Angiotensin II receptor type 1
BRET: Bioluminescence resonance energy transfer
CRTh2: Prostaglandin DP2 receptor
dcTMD: Dissipation-corrected targeted molecular dynamics
ECL2: Extracellular loop 2
EC_50_: Half-maximal effective concentration
FM: Funnel metadynamics
GaMD: Gaussian accelerated molecular dynamics
GPCRs: G protein-coupled receptors
gREST: Generalized replica exchange with solute tempering
Hsp90: Heat shock protein 90α
IC_50_: Half-maximal inhibitory concentration
iMetaD: Infrequent metadynamics
K_D_: Dissociation constant
*K*_*i*_: Inhibition constant
k_on_: Association rate constant
k_off_: Dissociation rate constant
MCK: Multi-cycle kinetics
MD: Molecular dynamics
MetaMD: Metadynamics
MSM: Markov state modeling
MST: Microscale thermophoresis
NTS1B: Neurotensin receptor B
PES: Potential energy surface
RAMD: Random acceleration molecular dynamics
REMD: Replica exchange molecular dynamics
REUS: Replica-exchange umbrella sampling
RMSD: Root mean square deviation
RT: Residence time
SBP: Secondary binding pocket
SCK: Single-cycle kinetics
sEH: Soluble epoxide hydrolase
sMD: Scaled molecular dynamics
SPR: Surface plasmon resonance
ssMD: Selective scaling molecular dynamics
TIS: Transition interface sampling
TM: Transmembrane helix
TMD: Targeted molecular dynamics
TPS: Transition path sampling
TRF: Time-resolved fluorescence
TR-FRET: Time-resolved fluorescence resonance energy transfer
TSPO: Translocator protein
WT-MD: Well-tempered metadynamics
